# Louisville Medical Institute

**Published:** 1845-09

**Authors:** 


### LOUISVILLE MEDICAL INSTITUTE.

From an advertisement on the cover it will be seen, that the regular term of lectures in the Medical Institute will commence on the first Monday in November. It should be borne in mind by students that after one introductory lecture the didactic course is begun, and that it is therefore important to be present at the beginning. From the number of students now in the city, and the number who have made arrangements for attending the Fall course of lectures, it is believed that the next class in the Institute will be very large.

